# SWI/SNF Subunits SMARCA4, SMARCD2 and DPF2 Collaborate in MLL-Rearranged Leukaemia Maintenance

**DOI:** 10.1371/journal.pone.0142806

**Published:** 2015-11-16

**Authors:** V. Adam Cruickshank, Patrycja Sroczynska, Aditya Sankar, Satoru Miyagi, Carsten Friis Rundsten, Jens Vilstrup Johansen, Kristian Helin

**Affiliations:** 1 Biotech Research and Innovation Centre (BRIC), University of Copenhagen, Ole Maaløes Vej 5, 2200 Copenhagen, Denmark; 2 Centre for Epigenetics, University of Copenhagen, Ole Maaløes Vej 5, 2200 Copenhagen, Denmark; 3 The Danish Stem Cell Centre (Danstem), University of Copenhagen, Blegdamsvej 3, 2200 Copenhagen, Denmark; Bellvitge Biomedical Research Institute (IDIBELL), SPAIN

## Abstract

Alterations in chromatin structure caused by deregulated epigenetic mechanisms collaborate with underlying genetic lesions to promote cancer. SMARCA4/BRG1, a core component of the SWI/SNF ATP-dependent chromatin-remodelling complex, has been implicated by its mutational spectrum as exerting a tumour-suppressor function in many solid tumours; recently however, it has been reported to sustain leukaemogenic transformation in MLL-rearranged leukaemia in mice. Here we further explore the role of SMARCA4 and the two SWI/SNF subunits SMARCD2/BAF60B and DPF2/BAF45D in leukaemia. We observed the selective requirement for these proteins for leukaemic cell expansion and self-renewal *in-vitro* as well as in leukaemia. Gene expression profiling in human cells of each of these three factors suggests that they have overlapping functions in leukaemia. The gene expression changes induced by loss of the three proteins demonstrate that they are required for the expression of haematopoietic stem cell associated genes but in contrast to previous results obtained in mouse cells, the three proteins are not required for the expression of c-MYC regulated genes.

## Introduction

Epigenetic modifiers have gained attention as potential therapeutic targets in leukaemia, as novel DNA and histone modifications and the enzymes that establish or remove these modifications are discovered. Alterations in the epi-genome convey heritable gene expression patterns, which are often linked to leukaemia. DNMT3A [1,2], EZH2 [3–5] and TET2 [6–8] are examples of chromatin modifiers that have been associated with acute myeloid leukaemia. Some of these proteins are amenable to drug intervention as has been demonstrated by several recent publications, reviewed in Helin and Dhanak, 2013 [9]. In this context, it becomes relevant to question if candidate chromatin-modifying proteins have cell-type specific functions in order to advance them as potentially relevant drug targets.

In human acute myeloid and lymphoid leukaemia driven by MLL rearrangements (MLLr), transcriptional elongation is taking the centre stage in explaining the molecular mechanism of sustained transformation via increased transcriptional rate at select loci coding for proteins that are involved in the initiation or maintenance of the transformed state. Indeed, both inhibition of P-TEFb associated CDK9 by flavopiridol and BRD4 eviction from chromatin by JQ1 and I-BET151 diminish *c-Myc* and *Hoxa9* expression and abolish self-renewal potential of MLLr-driven leukaemia [10–13].

MLL rearrangements lead to loss of the methyltransferase activity of MLL and the C-terminal portion of the translocation partner (most frequently AF9, AF4 and ENL in AML) recruits the fusion protein to complexes associated with transcriptional elongation (reviewed in Deshpande *et al*., 2012 [14]). Within this context, loci need to be accessible to the transcriptional machinery, thus entailing a possible role of the ATP-dependent chromatin remodeler family of genes in modifying the chromatin landscape and in this way contributing to disease.

The SWI/SNF chromatin remodelling complexes have been for the greater part implicated as exerting a tumour-suppressor function in solid tumours, including ovarian [15], hepatocellular [16] and renal carcinomas [17] as well as in pancreatic cancer [18]. Also, analyses of many sequencing studies have shown that SWI/SNF components are mutated in approximately 20% of human malignancies [19] (reviewed in Wilson et al., 2011 [20]). *Smarca4* ^*-/-*^ (Brg1, Snf2b) mouse embryos die at the peri-implantation stage while *Smarca4* ^*+/-*^ mice are indeed predisposed to tumour development [21]. This is similar to the knockout of SWI/SNF component *Snf5*/*Ini*, where homozygous embryos are unviable and heterozygote mice are prone to sarcomas and rhabdoid tumours [22,23]. One study however, shows that the tumorigenic mechanism underlying SNF5 mutation is not due to SWI/SNF inactivation, but is reliant on residual SMARCA4 (BAF190, BRG1, SNF2) activity in those cells [24].

In this study, we validate three SWI/SNF components: Smarca4, Smarcd2 and Dpf2, highly ranking in our previously published RNAi-based screen for genes required for the proliferation of MLL-AF9 transformed haematopoietic cells [25] as being required for leukaemic cell proliferation. These results are largely in agreement with a recent report by Vakoc and colleagues [26], however we report lack of *Myc* gene expression changes upon depletion of the SWI/SNF subunits.

## Results

### Depletion of single SWI/SNF components inhibits AML maintenance

**Fig 1:**
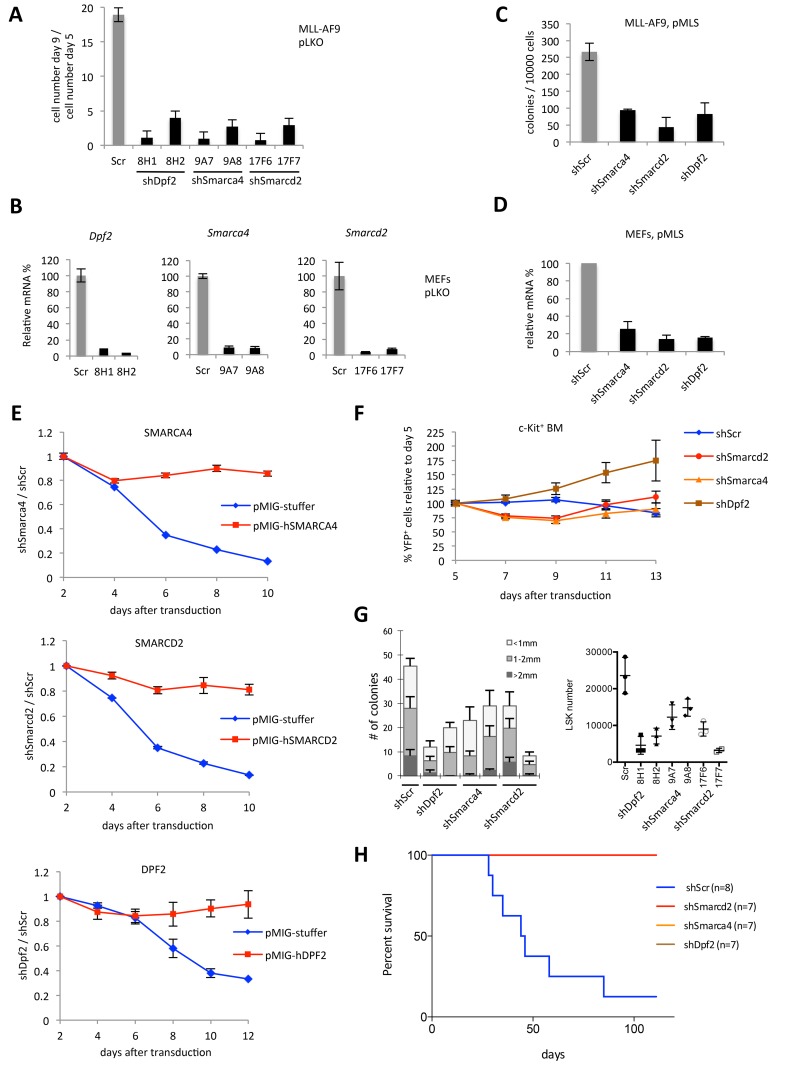
Depletion of single SWI/SNF complex components inhibits AML maintenance. (A) Mouse MLL-AF9 cell number fold change between day 5 and day 9 after transduction. Cells were transduced with pLKO constructs expressing the indicated shRNAs and selected with puromycin from day 2 after transduction. (B) Relative mRNA levels of *Smarca4*, *Smarcd2* and *Dpf2* in MEFs transduced with the indicated pLKO constructs. (C) Number of colonies generated by MLL-AF9 cells transduced with the indicated pMLS vectors. (D) Relative mRNA levels of *Smarca4*, *Smarcd2* and *Dpf2* in MEFs transduced with the indicated pMLS vectors. (E) Rescue experiments. MLL-AF9 cells were co-transduced with pMLS-YFP carrying shScr, shSmarca4, shSmarcd2 or shDpf2, as indicated, and control pMIGRI (pMIG-Stuffer) or pMIGRI expressing human *SMARCA4*, *SMARCD2* or *DPF2* cDNA. Normalized ratios of GFP^+^/YFP^+^ cell percentages between shSmarca4, shSmarcd2 or shDpf2 and shScr samples are plotted over a time course starting from day 2 after transduction. (F) Competitive proliferation assay of c-Kit-enriched mouse bone marrow cells transduced with the indicated pMLS vectors. (G) Left: Number of colonies generated by LSK cells transduced with the indicated pLKO shRNAs. Right: Absolute numbers of LSK cells with indicated knockdown in liquid culture. (H) Survival curves of sublethally irradiated mice transplanted with 10^4^ MLL-AF9 cells transduced with the indicated pMLS vectors.

In order to assess the effect of SWI/SNF complex subunits Smarca4, Smarcd2 or Dpf2 depletion in leukaemia, we transduced MLL-AF9 transformed mouse spleen cells with viruses expressing shRNA target sequences against the genes of each subunit. Cell numbers were similarly decreased with depletion of each subunit as soon as 4 days after initial puromycin selection (Fig 1A) and the degree of inhibition correlated with the knockdown efficiency of the tested shRNAs (Fig 1B). Similarly, the clonal-expansion capacity was hindered by downregulating the three SWI/SNF subunits when compared to cells transduced with control virus (shScr, Fig 1C and 1D). In support of these observations, forced expression of the human *SMARCA4*, *SMARCD2* or *DPF2* cDNA in mouse cells expressing mouse-specific shRNAs was able to rescue the cell-proliferation defect (Fig 1E).

The expression of the same constructs used for knockdown in transformed MLL-AF9 cells were unable to cause a proliferation defect on primary c-Kit-enriched bone marrow cells in culture, suggesting a leukaemia-specific requirement of these SWI/SNF complex components (Fig 1F). Knockdown of these subunits did, however, reduce the number of colonies generated from Lin^-^ Sca1^+^ cKit^+^ (LSK) cells as well as LSK cell proliferation (Fig 1G).

To assess the relevance of our observations in a physiological context, we transplanted sublethally-irradiated mice with MLL-AF9 cells transduced with shRNAs targeting either *Smarca4*, *Smarcd2*, *Dpf2*, or non-targeting control (shScr) and monitored mice for the onset of leukaemia. As shown in Fig 1H, downregulation of the SWI/SNF complex subunits significantly extended overall survival, with all 21 mice surviving until the termination of the experiment.

### Inhibition of *Smarca4*, *Smarcd2* or *Dpf2* expression leads to apoptosis, cell cycle changes and myeloid differentiation in leukaemic cells

**Fig 2:**
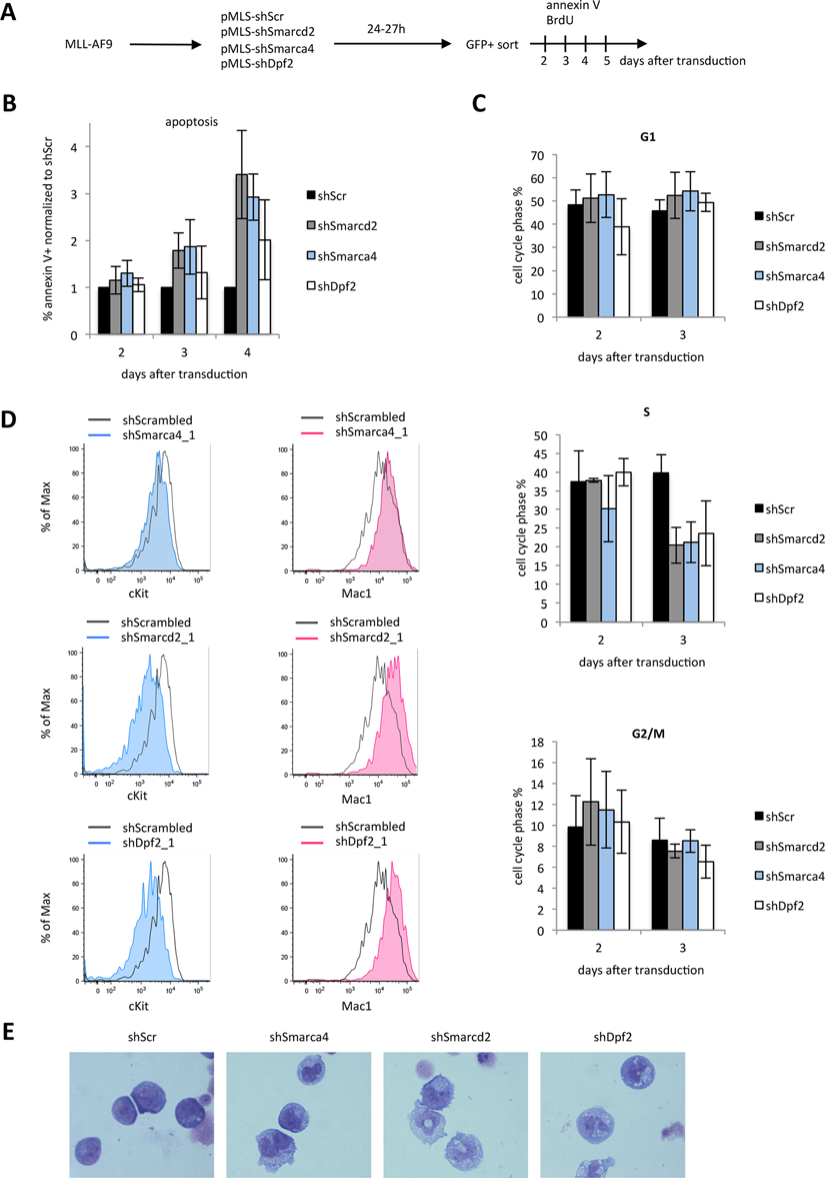
Inhibition of Smarca4, Smarcd2 or Dpf2 expression leads to apoptosis, cell cycle changes and myeloid differentiation. (A) Experimental overview for panels B and C. (B) Relative percentage of Annexin V-positive MLL-AF9 cells at days 2, 3 and 4 after transduction with non-targeting control (shScr) or shRNAs targeting *Smarcd2*, *Smarca4* and *Dpf2*. (C) Cell cycle analysis of Smarcd2, Smarca4 and Dpf2-depleted MLL-AF9 cells compared to control cells (shScr). (D) Flow cytometry analysis of c-Kit and Mac1 staining in MLL-AF9 cells transduced with shScr, shSmarca4, shSmarcd2 or shDpf2. (E) Representative images of May-Grünwald-Giemsa stained MLL-AF9 cells transduced with the indicated shRNAs.

To investigate the growth defect caused by SWI/SNF-complex subunit depletion, we measured the fraction of apoptotic cells (Fig 2A). Mouse MLL-AF9 cells with knockdown of SWI/SNF-complex subunits displayed increasing apoptosis over time as compared to shScr (Fig 2B). Onset of apoptosis was observed subsequent to a reduction in the percentage of cells in S-phase (Fig 2C).

To investigate whether the delay in cell cycle progression is due to terminal differentiation, we stained mouse MLL-AF9 cells 4 days after transduction with antibodies to c-Kit and Mac1/Cd11b (a myeloid marker) and analysed them by flow-cytometry. The expression of the haematopoietic stem and progenitor cell (HSPC) marker c-Kit was decreased in cells in which the expression of Smarca4, Smarcd2 or Dpf2 was downregulated when compared to control (Fig 2D). In contrast, the expression of Mac1 was increased in the knockdown populations with respect to control. In addition, May-Grünwald-Giemsa staining revealed an increased frequency of monocytic and granulocytic cell morphologies in knockdown cell-populations (Fig 2E). Together these results indicate that each of the invesigated SWI/SNF subunits, Smarca4, Smarcd2 and Dpf2, has a role in maintaining the undifferentiated state of MLLr-transformed blasts.

### SMARCA4, SMARCD2 and DPF2 maintain leukaemic self-renewal independently of *MYC* transcription status in human cells

**Fig 3:**
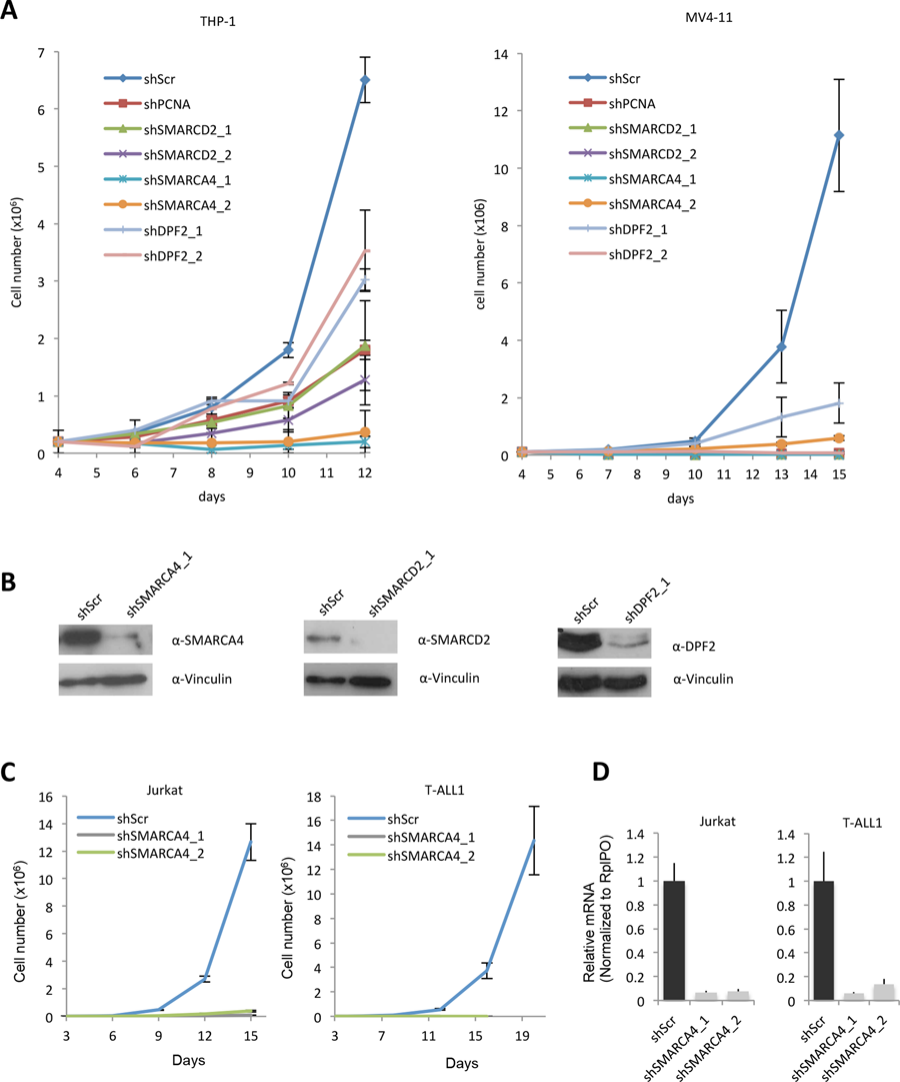
Knockdown of SWI/SNF complex subunits impairs growth of human leukaemic cells (A) Number of THP-1 and MV4-11 cells over a time course starting from day 4 after transduction. Cells were transduced with the indicated pLKO vectors. (B) Western blots showing SMARCA4, SMARCD2 and DPF2 levels in THP-1 cells transduced with shScr or the indicated pLKO vectors. Vinculin was used as a loading control. (C) Number of Jurkat and T-ALL1 cells over a time course starting from day 3 after transduction. Cells were transduced with the indicated pLKO vectors. (D) Relative mRNA levels of *SMARCA4* in Jurkat and T-ALL1 cells transduced with the indicated pLKO constructs.

To explore the transcriptional effect of SWI/SNF subunit depletion in the human cell lines, we first assessed the effect of SWI/SNF subunit knockdown on proliferation of MLL-AF9 (THP-1) and MLL-AF4 (MV4-11) driven myeloid leukaemia (Fig 3A). SMARCA4, SMARCD2 or DPF2 depletion in THP-1 cells (Fig 3B), leads to a marked growth defect of cells in culture. Importantly, knockdown of any of the tested subunits does not affect the expression of the two other subunits (S1 Fig). Human T-cell acute lymphoid leukaemia derived Jurkat and T-ALL1 cells also require full SMARCA4 expression for unperturbed proliferation *in-vitro* (Fig 3C and 3D).

**Fig 4:**
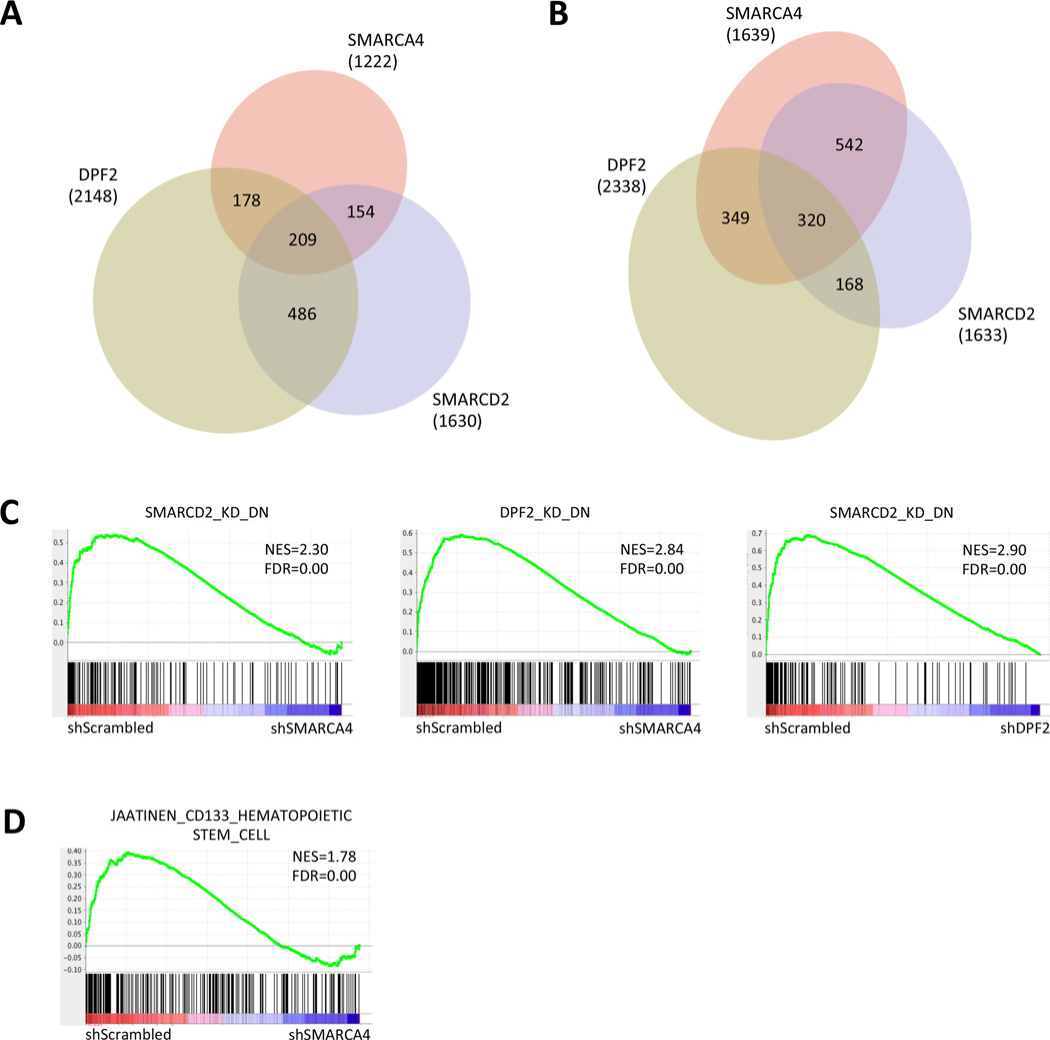
Gene expression changes upon knockdown of single SWI/SNF complex components in human AML cells. (A and B) Venn diagrams showing overlap of genes significantly changing down (A), or up (B) (p<0.05) upon SMARCA4, SMARCD2 and DPF2 knockdown in THP-1 cells. (C-D) Gene set enrichment analysis (GSEA) plots showing enrichment of indicated gene sets in genes ranked by Signal2noise metric in SMARCA4 or DPF2 knock-down versus control THP-1 cells.

Next we determined the global transcriptional changes induced by the diminished levels of each of the three subunits in THP-1 cells. A low, but significant fraction of genes with increased expression (320, 5.7%, p<0.01) or with decreased expression (209, 4.18%, p<0.01) were found to overlap with all three subunits (Fig 4A and 4B). To further explore these changes, we defined SMARCA4, SMARCD2 and DPF2_UP/DOWN gene sets comprised of genes changing at least 2 fold (FDR<0.05) in each case, and used gene set enrichment analysis (GSEA) to query the enrichment of one gene set in the most changing microarray data of another subunit. Indeed, each of these two-way comparisons showed a highly significant overlap (p<0.01, normalised enrichment scores NES > [2.3]) suggesting a cooperative function of these three subunits at transcriptional level in leukaemia (Fig 4C and S2 Fig). In addition, SMARCA4 knockdown correlates with the loss of a haematopoietic stem cell signature defined in human CD133^+^ cord blood cells [27], implicating the role for SMARCA4 in self-renewal maintenance (Fig 4D).

**Fig 5:**
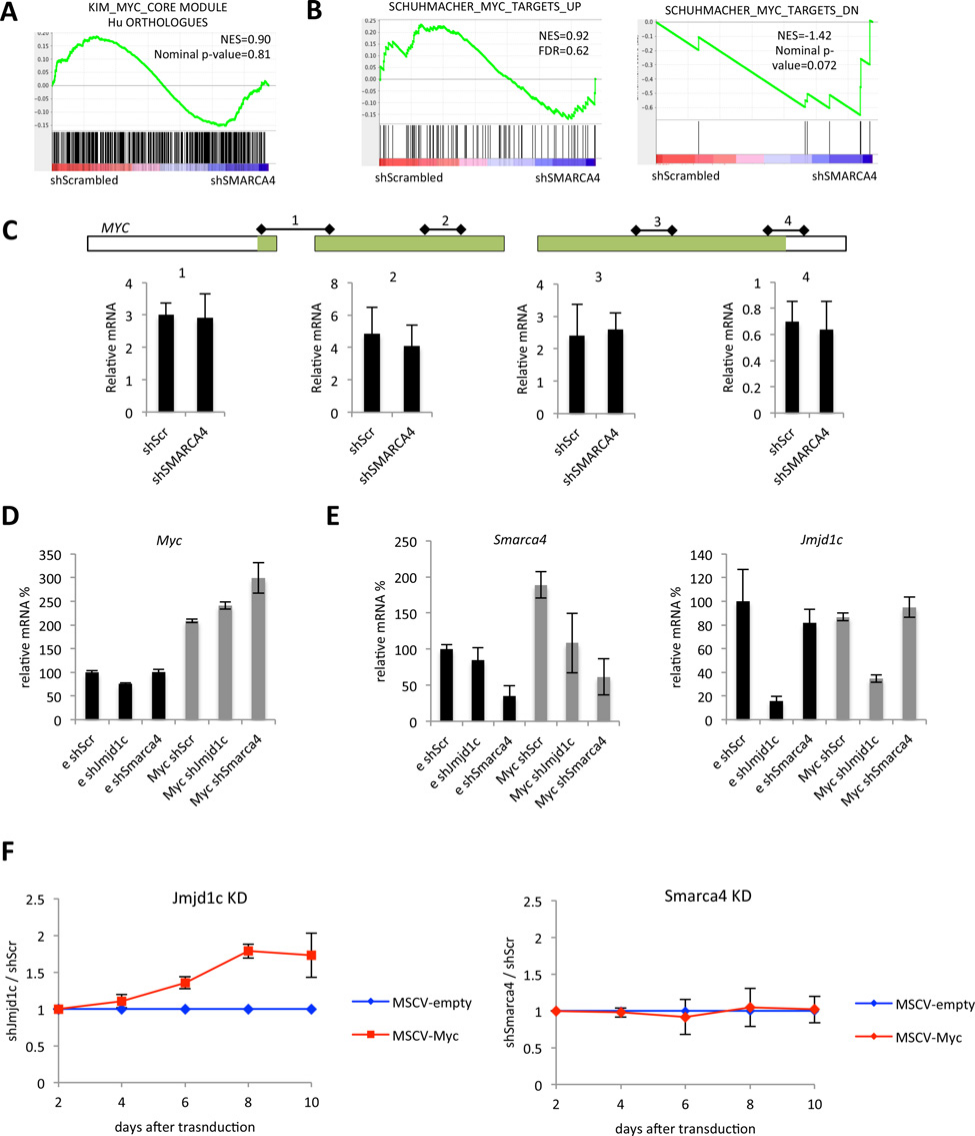
No changes in *MYC* expression after SMARCA4 knockdown. (A-B) GSEA plots showing enrichment of the indicated gene sets in genes ranked by Signal2noise metric in SMARCA4 knockdown versus control THP-1 cells. (C) Top: schematic representation of the human *MYC* locus; coding sequence is marked in green; qPCR primer pairs and their amplification products are represented as regions 1–4. Bottom: Relative mRNA levels of *MYC* assessed by using the indicated primer pairs. (D-F) Myc rescue experiments of Smarca4 and Jmjd1c KD in MLL-AF9 cells. (D-E) Relative mRNA levels of the indicated genes in cells transduced with the indicated combinations of vectors. e, empty vector; Myc, Myc expression vector; shScr, non-targeting control pMLS-YFP vector; shJmjd1c and shSmarca4, pMLS-YFP vectors targeting Jmjd1c and Smarca4, respectively. (F) Normalised ratios of GFP^+^ YFP^+^ cell percentages between shJmjd1c and shScr samples (left) or shSmarca4 and shScr samples (right) plotted over an 8-day time course starting from day 2 after transduction.

A recent study suggested a role for Smarca4 in sustaining Myc-dependent leukaemic transformation in mouse AML induced by MLL-AF9 and NRASG12D [26]. We did not, however, detect a loss of a c-MYC transcription factor-defined embryonic stem cell module [28] (Fig 5A) nor of one defined in *c-MYC*-dependent B cells [29] (Fig 5B). We also failed to observe any significant change in *c-MYC* transcript levels with *SMARCA4* knockdown in our microarray data (S1 Table) or by RT-qPCR in THP-1 or in mouse MLL-AF9 transformed cells (Fig 5C and 5D). We also failed to achieve any rescue of the cell proliferative defect caused by *Smarca4* knockdown in MLL-AF9 cells with *c-Myc* overexpression with a *c-Myc* cDNA that complemented RNAi against Jmjd1c (Fig 5E and 5F), which was used as a positive control [25].

## Discussion

In this study we explore the requirement of three SWI/SNF associated proteins in leukaemia maintenance. SMARCA4 and SMARCA2 are two mutually exclusive ATP-dependent helicases of SWI/SNF complexes. Despite these subunits being structurally almost identical, several functional differences are apparent; mouse *Smarca2* is dispensable during embryonic development whereas *Smarca4* ^-/-^ embryos die at pre- or peri-implantation stage [21]. Different expression patterns in a variety of normal tissues also support the notion of these ATPase subunits carrying out separate functions [30].

SMARCD2 co-purifies with SMARCA4 [31] and contains a SWIB/MDM2 domain [32]. DPF2 is a double plant homeodomain (PHD) finger protein that associates with members of the SWI/SNF complex and mediates binding to acetylated histone H3 [33]. By examining the effect of *Smarca4*, *Smarcd2* or *Dpf2* depletion in mouse MLL-AF9 cells, we demonstrated that the three proteins are required for their proliferation and self-renewal. The diminished proliferation was associated with cell cycle perturbations and increased terminal differentiation and apoptosis. Importantly, the depletion of any of these SWI/SNF complex subunits extended survival of mice transplanted with leukaemic cells, thereby demonstrating the *in-vivo* relevance of our findings.

Mouse c-Kit-enriched bone marrow (BM) cells did not display any growth impairment, but when depleting *Smarca4*, *Smarcd2* or *Dpf2* in the LSK cell subpopulation we observed a hindrance of both proliferation in liquid culture and colony formation capacity. The similarity between the effect of depletion of each of these subunits suggest that they work as a complex in AML, which is in agreement with two recent publications [26] and [34]. Indeed, gene expression analysis in the human MLL-AF9-derived myeloid leukaemia cell line THP-1 revealed a three-way overlap between both up- and down-regulated genes with the individual depletion of each subunit. Despite this overlap not being major and therefore revealing many possible SMARCA4-independent roles for SMARCD2 and DPF2, additional two-way GSEA comparisons between most changing genes (0.5>FC>2, FDR<0.05) in each case further support the notion of these three subunits indeed acting through the same mechanism. This mechanism however, appears to be *c-MYC* independent, given that *c-MYC* expression levels are not altered in these cells. This stands in contrast to a recent study that convincingly showed the implication of a cell type-specific enhancer region downstream of the *c-Myc* promoter in driving increased *c-Myc* expression in a Smarca4-dependent manner in mouse cells [26]. Analysis of the expression changes imposed by downregulation of *SMARCA4* in human THP-1 cells, however, showed that inhibition of *SMARCA4* expression does not affect Myc-dependent gene expression signatures, previously defined in ES cells [28] and in *c-MYC*-dependent B cells [29]. There was a significant enrichment of a gene set defined by genes up-regulated in CD133^+^ cord blood cells [27] that was lost with *SMARCA4* knockdown, supporting a role for SMARCA4 in self-renewal.

The mouse leukaemic (RN2) cells used throughout the study performed by Shi et al. [26] were generated by over-expression of both the MLL-AF9 fusion protein and a constitutively active mutant form of NRAS (NRASG12D). By contrast, the cells used in this study do not express NRASG12D. NRAS mutations are significantly underrepresented in the AML patient subgroup with MLL-rearrangements (3 out of 77; 3.9%; p = 0.061) [35] and no significant prognostic impact of mutated NRAS was found for overall, event-free, or disease-free survival. We therefore predict that NRASG12D mutations have a minor role in the underlying mechanism of SWI/SNF dependent leukaemia.

## Methods

### Cell culture

Mouse primary MLL-AF9 AML cells were generated during a previous study [25]. Culture and transduction of MLL-AF9 AML, c-Kit^+^ BM and LSK cells were done as previously described [25]. THP-1 and MV4-11 cells were obtained from DSMZ (http://www.dsmz.de/home.html) and were cultured according to supplier’s protocol. Transduction was done with retro- or lentivirus using the RetroNectin®-bound Virus (RBV) infection method according to manufacturer’s protocol (Takara). Selection was with 2 μg/ml of puromycin for 48–72 hours prior to start of assay and then maintained. For colony forming assays, methylcellulose media M3534 and M3434 were used (for MLL-AF9 and LSK respectively, STEMCELL Technologies).

### Virus production

For retrovirus production, Phoenix-Ecotropic cells [36] were cotransfected with pMLS or pMSCV vectors and pCL-Eco by using a calcium phosphate transfection method. For lentivirus production, 293FT cells were cotransfected with pLKO.1-puro (Sigma- Aldrich), pAX8 and pCMV-VSV by using a calcium phosphate transfection method [37].

### Mouse transplantation

For secondary transplants, shSmarca4-, shSmarcd2-, shDpf2- or shScr-pMLS-transduced MLL-AF9 spleen leukaemic cells were FACS sorted two days after infection and injected into sublethally irradiated (450 cGy) B6.SJL recipient mice at 10^4^ cells per recipient by tail-vain injection. There were 7 or 8 mice in each group. No signs of post-irradiation sickness were observed at any point during this study. Mice were moniotred at least every other day and euthanized by cervical dislocation as soon as any of the following signs of advanced leukaemia were observed: paleness, hunched posture, laboured breathing or loss of activity. The study was approved by the Danish Animal Protection Agency (*Dyreforsøgstilsynet*) under the license number 2011/561−1972. The protocol for mouse survival curves was approved by the Department of Experimental Medicine (*Afdeling for Eksperimentel Medicin*, *AEM*) of the University of Copenhagen under the project number P13-018, valid through 2013 and 2014.

### Messenger RNA expression analysis

RNA was purified by using an RNeasy Plus RNA kit (Qiagen) and reverse transcribed by using TaqMan Reverse Transcription Reagents (Applied Bio-systems). Quantitative reverse transcription PCR (qRT-PCR) was performed with LightCycler 480 SYBR Green I Master and a LightCycler480 System (Roche). mRNA levels were normalized to *Rplp0*. Primer sequences can be found in Supporting Information.

### Ectopic cDNA expression

cDNA clones were purchased at Source BioSciences http://www.lifesciences.sourcebioscience.com and cloned into pCR®8/GW/TOPO® TA, sequenced and subcloned into pMIGRI-flag expression vectors by Gateway® cloning. Clone IDs and sequences of cloning primers can be found in Supporting Information.

### Flow cytometry and cell sorting

Cells were sorted using BD FACSAriaI or BD FACSAriaIII cell sorters. Extracellular marker expression, apoptosis and cell cycle analysis were performed as described elsewhere [25].

### Western blotting

Primary antibodies used are: anti-SMARCA4 (Millipore 3G4), anti-SMARCD2 (Santa Cruz, F-19 102119), anti-DPF2 (Sigma, HPA020880), or anti-Vinculin (Sigma, V9131). Secondary: Peroxidase labelled anti-rabbit, anti-rat or anti-mouse IgG (Vector).

### May-Grünwald-Giemsa staining

Cells were spun onto glass slides, air-dried, submerged in May-Grünwald and Giemsa staining solutions (Merck) and rinsed in phosphate buffer pH 6.5 (Ampliqon). Images were acquired at 100X magnification.

### Expression microarray

RNA was extracted with an RNeasy Plus RNA kit (Qiagen) and hybridized on Agilent SurePrint G3 Human GE v2 8x60K Microarray according to the manufacturer’s protocol. GEO accession number: GSE67924.

### Gene set enrichment analysis (GSEA)

GSEA (http://www.broadinstitute.org/gsea/index.jsp) was performed as described [25]. Briefly, knockdown (KD) vs. scrambled (Scr) were run from triplicate expression files. Gene sets used are listed in S2 Table and at http://www.broadinstitute.org/gsea/msigdb/index.jsp. For all gene sets, 1000 permutations and the Signal2Noise metric were used. Permutations by gene sets were conducted to assess statistical significance.

## Supporting Information

S1 Fig Western blot analysis of SMARCD2, SMARCA4 and DPF2 levels in THP-1 cells transduced with shScr or the indicated pLKO vectors. Vinculin was used as a loading control.
(TIF)

S2 Fig GSEA plots showing enrichment of the indicated gene set in genes ranked by Signal2noise metric in SMARCA4 knockdown versus control THP-1 cells. (TIF)

S1 Table THP-1 microarray. 4C3, shSMARCA4; 4D1, shSMARCD2; 8E6, shDPF2.
(XLS)

S2 Table Gene sets used in this study. http://www.broadinstitute.org/gsea/msigdb/index.jsp
(XLSX)
