# Supplementary figures and images for: SWI/SNF Subunits SMARCA4, SMARCD2 and DPF2 Collaborate in MLL-Rearranged Leukaemia Maintenance
Source: PLoS One. 2015 Nov 16;10(11):e0142806. doi: 10.1371/journal.pone.0142806 (PMC4646637; doi:10.1371/journal.pone.0142806)

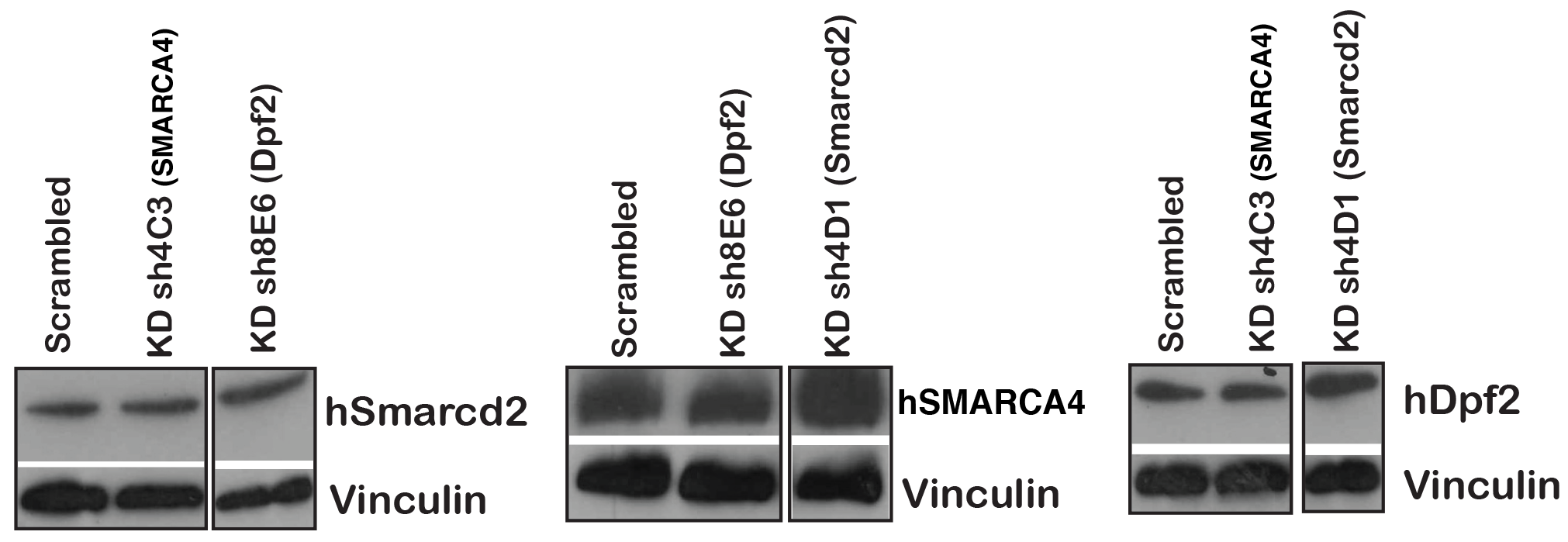

Supplement: S1 Fig — Vinculin was used as a loading control. (TIF) [file pone.0142806.s001.tif]

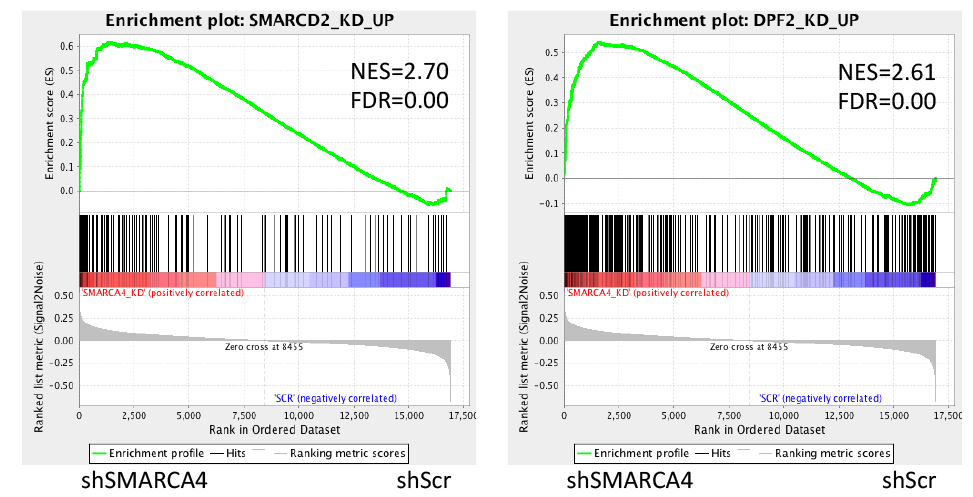

Supplement: S2 Fig — (TIF) [file pone.0142806.s002.tif]
